# Studies on 2-Arylhydrazononitriles: Synthesis of 3-Aryl-2-arylhydrazopropanenitriles and Their Utility as Precursors to 2-Substituted Indoles, 2-Substituted-1,2,3-Triazoles, and 1-Substituted Pyrazolo[4,3-*d*]pyrimidines

**DOI:** 10.3390/molecules171012225

**Published:** 2012-10-18

**Authors:** Khaled D. Khalil, Hamad M. Al-Matar

**Affiliations:** 1 Chemistry Department, Faculty of Science, University of Kuwait, P.O. Box 5969, Safat 13060, Kuwait; Email: h.almatar@ku.edu.kw; 2 Chemistry Department, Faculty of Science, Cairo University, Giza, 12613, Egypt

**Keywords:** benzylidenemalononitrile, 2-arylhydrazononitrile, amidoxime, cyanoindole, dimethylformamide dimethylacetal, 1,2,3-triazole

## Abstract

Coupling of 2-benzylmalononitrile with aromatic diazonium salts afforded 3-phenyl-2-arylhydrazonopropanenitriles **4a**,**b**, which were rearranged into 2-cyanoindoles **5a**,**b** upon heating with ZnCl_2_ in the presence of glacial acetic acid. The produced indole derivatives **5a**,**b** can be successfully used as valuable precursors to synthesize 1,2,4-oxadiazolylindoles **8a**,**b**. The reaction of arylhydrazononitriles **4a**,**b** with hydroxylamine afforded an amidoximes **9a**,**b** that could be cyclized into 1,2,3-triazole-4-amines **10a**,**b**. In addition, **4a**,**b** could be converted into 4-aminopyrazoles **12a**,**b** via condensation with chloroacetonitrile in the presence of triethylamine as a basic catalyst. Finally, compounds **12a**,**b** were refluxed with dimethylformamide dimethylacetal (DMFDMA) to afford amidines **13a**,**b** that were readily cyclized to the corresponding pyrazolo[4,3-*d*]pyrimidines **14a**,**b** when refluxed with ammonium acetate.

## 1. Introduction

2-Arylhydrazononitriles **4a**,**b** are versatile reagents and their chemistry has recently attracted considerable interest [1,2,3,4,5,6,7,8]. In previous recent work we have established the utility of these compounds as precursors for 1,2,4-triazoles [5], 1,2,3-triazoles [6], and pyrazolo[1,5-*a*]pyrimidines [7,8]. In conjunction to that work we report herein an easy route to the title compounds and their utility as precursors for synthesis of various heterocycles. 1,2,3-Triazine derivatives are an important class of heterocyclic compounds that are considered useful precursors in organic synthesis and as pharmaceuticals (e.g., as antimalarials) [9,10,11]. In this article, we enabled development of an easy approach to 1,2,4-oxadiazolylindole [12,13], and pyrazolo[4,3-*d*]pyrimidine derivatives of notable biological and pharmaceutical importance [14,15,16].

## 2. Results and Discussion

**Scheme 1:**
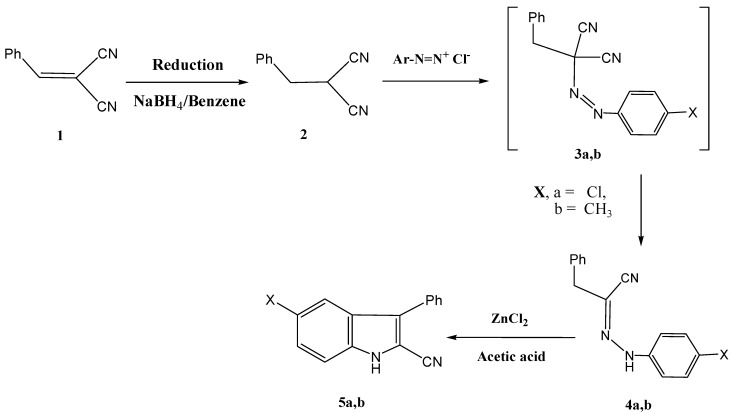
Synthesis of indole-2-carbonitriles **5a**,**b**.

The hydrazononitriles **4a**,**b** were synthesized by reducing benzylidenemalononitrile (**1**) with sodium borohydride as recently described, to yield **2** [17]. Coupling of compound **2** with aromatic diazonium salts afforded intermediates **3**. It is believed that the initially formed **3a**,**b** readily undergo Japp-Klingmann cleavage [18] yielding the final isolable products **4a**,**b** in 75%, and 70% yield respectively. Compounds **4a**,**b** afforded the 2-cyanoindoles **5a**,**b** upon treatment with zinc chloride and glacial acetic acid. This is an example of the utility of the Fisher indole synthesis in the synthesis of 2-cyanoindoles (Scheme 1).

**Scheme 2:**
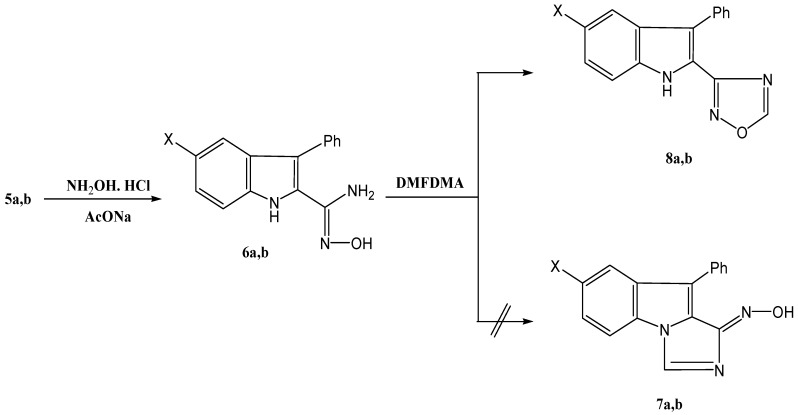
Synthesis of 1,2,4-oxadiazolylindole derivatives **8a**,**b**.

The 3-phenylindole-2-carbonitriles **5a**,**b** reacted with hydroxylamine hydrochloride to yield amidoximes **6a**,**b**. Reacting these products with dimethylformamide dimethylacetal (DMFDMA) afforded products **8a**,**b** in 68%, and 65% yield respectively, rather than **7**, as indicated by a NOE experiment that showed an interaction between the indole-H-1, at 10.4 ppm and indole-H-7, at 6.8–7.3 ppm (Scheme 2).

**Scheme 3:**
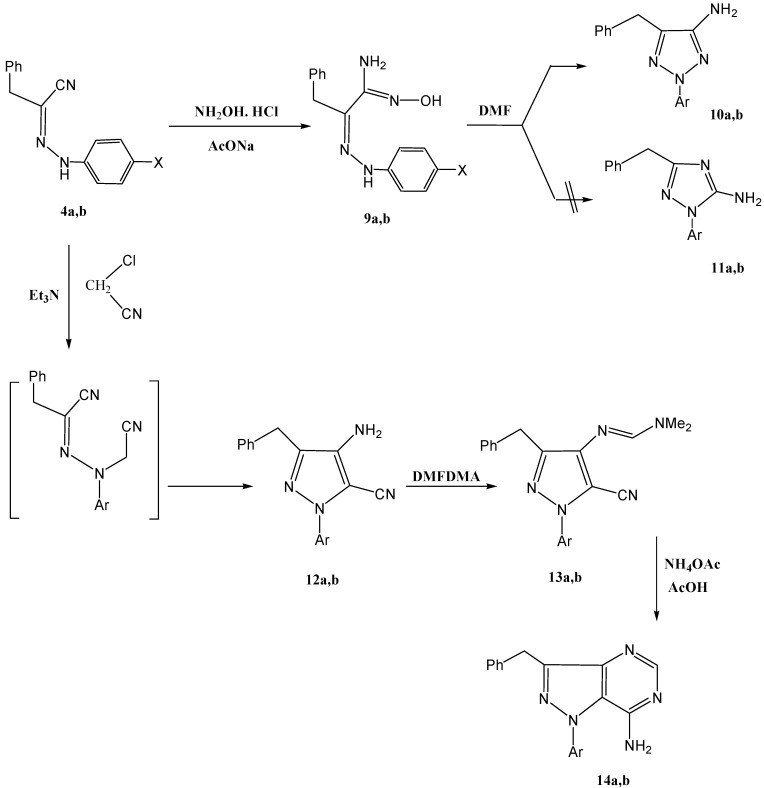
Synthesis of 1,2,3-triazoles and pyrazolo[4,3-*d*]pyrimidines.

Our attention then shifted to explore the utility of 2-arylhydrazonals as efficient precursors to 1,2,3-triazoles. Compounds **4a**,**b** reacted with hydroxylamine hydrochloride to yield amidoximes **9a**,**b** that could be cyclized into **10a**,**b** or the isomeric **11a**,**b** upon reflux in DMF. From the previously reported findings concerning this reaction, the structure of the product is not clear, where the 1,2,3-triazole **10a**,**b** found a parallel in results reported for similar reactions under similar conditions [1,2,3]. Although cyclization into isoxazoles has been reported by either refluxing of amidoximes in acidic medium [4] or refluxing an ester derivative of an amidoxime in dimethylformamide [19], cyclization to a 1,2,4-triazole via a Tiemann-like rearrangement has been reported by us in one case [5]. Structures **11a**,**b** could be excluded due to the absence of any interaction between the NH_2_ protons and the aryl protons in a NOE experiment (Scheme 3). Moreover, we successfully confirmed that the correct structures are the 1,2,3-triazoles **10a**,**b** based on the obtained single crystal X-ray crystallography results recently reported by our group [6].

Compounds **4a**,**b** was refluxed with chloroacetonitrile to yield **12a**,**b** that were then refluxed with DMFDMA to give the expected amidines **13a**,**b**. The amidines, so formed, were then cyclized in the presence of NH_4_OAc and glacial acetic acid to give pyrazolo[4,3-*d*]pyrimidines **14a**,**b** (*cf*. Scheme 3). The structure of the products **14a**,**b** was confirmed by the spatial interaction between the NH_2_ protons, at 5.87 ppm, and aryl protons, at 7.08–7.17 ppm, in the NOE experiment.

## 3. Experimental

### 3.1. General Procedures

Melting points were recorded on a Gallenkamp apparatus and are uncorrected. Infrared spectra (KBr) were determined on a Jasco FT/IR-6300 FT-IR instrument. NMR measurements were determined on a Bruker DPX spectrometer at 600 MHz for ^1^H-NMR and 125 MHz for ^13^C-NMR, in DMSO-*d*_6_ as solvent and using TMS as internal standard. Mass spectra were measured on GC MS DFS-hermo spectrometers. Elemental analyses were measured by means of an Elementar Vario Micro Cube. Microwave heating was carried out with a single mode cavity Explorer Microwave Synthesizer (CEM Corporation, NC, USA), producing continuous irradiation and equipped with simultaneous external air−cooling system.

### 3.2. Synthesis of 2-Benzylidenemalononitrile *(**2**)*

This was prepared by the literature procedure [17]. A mixture of benzaldehyde (10 mmol) and malononitrile (0.66 g, 10 mmol) was dissolved in aqueous ethanol (1:4, 25 mL) and stirred overnight. The reaction was followed to completion by TLC. To the pre-cooled reaction mixture, an equivalent amount of NaBH_4_ was added portionwise with stirring at 0 °C for 15 min. The mixture was acidified with aqueous HCl and the product was extracted with CH_2_Cl_2_. The clear filtrate was evaporated under reduced pressure, and the remaining solid was collected by filtration. The solid product was then recrystallized from ethanol to give a colorless powder (82%); mp 85–86 °C (lit. mp 86–88 °C [20]); IR (KBr): *υ* = 2188.4 (CN), 2198 (CN) cm^–1^; ^1^H-NMR: *δ* = 3.28 (d, *J* = 7.0 Hz, 2H), 3.88 (t, *J* = 7.0 Hz, 1H), 7.32–7.44 (m, 5H, phenyl); ^13^C-NMR: *δ* = 25.2 (CH), 37.1 (CH_2_), 112.0 (2 CN), 128.6, 129.0, 129.2, 132.8 (aromatic carbons); MS, *m/z* (%): 156.07 (M^+^, 100), 77 (53); Anal. Calcd. for C_10_H_8_N_2_: C, 76.90; H, 5.16; N, 17.94. Found: C, 76.77; H, 5.09; N, 17.72.

*Coupling of* **2** *with aryldiazonium chlorides.* A cold solution of the appropriate aryldiazonium salt was prepared by adding sodium nitrite solution (1.4 g dissolved in 10 mL water) to a pre-cooled solution of the corresponding arylamine hydrochloride (*p*-chloroaniline or *p*-toluidine, 10 mmol of arylamine in 6 mL 6 M HCl) with continuous stirring. The resulting aryldiazonium salt solutions were then added carefully to a cold ethanolic solution (50 mL) of benzylidenemalononitrile (**2**, 10 mmol) and sodium acetate trihydrate (2.8 g, 20 mmol). The mixture was stirred at room temperature for 1 h and the solid product formed was collected by filtration, washed with water and recrystallized from ethanol.

*2-[(4-Chlorophenyl)hydrazono]-3-phenylpropionitrile* (**4a**). This compound was obtained as pale yellow solid (75%); mp ~148 °C; IR (KBr): *υ* = 3300 (br. NH), 2185 (CN) cm^–1^; ^1^H-NMR: *δ* = 2.61 (s, 2H, CH_2_), 7.02 (d, 2H, *J* = 8 *Hz*, aryl), 7.34 (d, 2H, *J* = 8 *Hz*, aryl), 7.44 (m, 5H, phenyl), 8.9 (s, 1H, NH); ^13^C-NMR: *δ* = 30.2 (CH_2_), 113.6, 117.6 (CN), 119.5, 125.3, 126.8, 128.0, 129.8, 133.0, 135.9 (aromatic carbons), 159.1 (C=N); MS, *m/z* (%): 269.1 (M^+^, 100), 77 (66); Anal. Calcd. for C_15_H_12_ClN_3_: C, 66.79; H, 4.48; Cl, 13.14; N, 15.58. Found: C, 66.68; H, 4.40; Cl, 13.05; N, 15.46.

*3-Phenyl-2-(p-tolylhydrazono)propionitrile* (**4b**). This compound was obtained as a yellow solid (70%); mp ~126 °C; IR (KBr): *υ* = 3320 (br. NH), 2189 (CN) cm^–1^; ^1^H-NMR: *δ* = 1.71 (s, 3H, CH_3_), 2.66 (s, 2H, CH_2_), 7.13 (d, 2H, *J* = 8 *Hz*, aryl), 7.18 (d, 2H, *J* = 8 *Hz*, aryl), 7.22 (m, 5H, phenyl), 11.5 (s, 1H, NH); ^13^C-NMR: *δ* = 36.2 (CH_3_), 38.6 (CH_2_), 117.4 (CN), 118.9, 123.5, 127.3, 129.2, 132.4, 134.6 (aromatic carbons), 157.5 (C=N); MS, *m/z* (%): 249.31 (M^+^, 100), 77 (54). Anal. Calcd. for C_16_H_15_N_3_: C, 77.08; H, 6.06; N, 16.85. Found: C, 76.97; H, 5.98; N, 16.77.

*Cyclization of* **4a**,**b** *in the presence of ZnCl_2_ and glacial acetic acid.* A mixture of **4a**,**b** (10 mmol), zinc chloride (1.34 g, 10 mmol), and glacial acetic acid (50 mL) was refluxed and followed by TLC till completion after 24 h. The reaction mixture was poured into an ice/water mixture and the solid product, thus formed, was then collected by filtration and recrystallized from ethanol. 

*5-Chloro-3-phenyl-1H-indole-2-carbonitrile* (**5a**). This compound was obtained as a yellow solid (60%); mp ~212 °C; IR (KBr): *υ* = 3300 (br. NH), 2206 (CN) cm^–1^; ^1^H-NMR: *δ* = 7.03–7.26 (m, 8H, aryl & phenyl), 11.1 (s, 1H, NH); ^13^C-NMR: *δ* = 117.6 (CN), 119.8, 121.4, 122.9, 124.7, 128.4, 129.6, 130.5, 133.0, 137.9 139.1, 142.2 (aromatic carbons); MS, *m/z* (%): 252.05 (M^+^, 100), 77 (51); Anal. Calcd. for C_15_H_9_ClN_2_: C, 71.29; H, 3.59; Cl, 14.03; N, 11.09. Found: C, 71.15; H, 3.52; Cl, 13.84; N, 10.97.

*5-Methyl-3-phenyl-1H-indole-2-carbonitrile* (**5b**). This compound was obtained as a colorless solid (70%); mp ~168 °C; IR (KBr): *υ* = 3320 (br. NH), 2189 (CN) cm^–1^; ^1^H-NMR: *δ* = 1.81 (s, 3H, CH_3_), 6.87–7.33 (m, 8H, phenyl), 10.8 (s, 1H, NH); ^13^C-NMR: *δ* = 35.2 (CH_3_), 117.6 (CN), 120.6, 122.3, 122.6, 123.4, 127.0, 128.7, 129.4, 132.7, 132.8, 134.6, 139.8 (aromatic carbons); MS, *m/z* (%): 232.1 (M^+^, 100), 77 (48); Anal. Calcd. for C_16_H_12_N_2_: C, 82.73; H, 5.21; N, 12.06. Found: C, 82.68; H, 5.14; N, 11.95.

### 3.3. Synthesis of 1,2,4-Oxadiazolyl-indoles ***8a**,**b***

A mixture of **5a**,**b** (10 mmol), hydroxylamine hydrochloride (0.69 g, 10 mmol), and sodium acetate (3 g, 25 mmol) in ethanol (25 mL) was refluxed for 5 h. The reaction mixture was poured into ice/water with stirring while a yellow solid separated and was then collected by filtration. The crude product was refluxed with DMFDMA for 6 h. The pure products **8a**,**b** were purified by recrystallization from ethanol.

*3-(5-Chloro-3-phenyl-1H-indol-2-yl)-1,2,4-oxadiazole* (**8a**). Obtained as a pale yellow powder (68%); mp ~142 °C; IR (KBr): *υ* = 1586 (aromatic C=C) cm^–1^; ^1^H-NMR: *δ* = 6.84–7.31 (m, 9H, aromatic), 10.4 (s, 1H, NH, imidazole); ^13^C-NMR: *δ* = 112.3, 115.2, 121.2, 122.8, 123.4, 124.0, 127.9, 128.5, 129.1, 131.4, 133.8, 134.7, 137.9, 148.6 (aromatic carbons); MS, *m/z* (%): 295.1 (M^+^, 56), 77 (100); Anal. Calcd. for C_16_H_10_ClN_3_O: C, 64.98; H, 3.41; Cl, 11.99; N, 14.21. Found: C, 64.91; H, 3.36; Cl, 11.91; N, 14.13.

*3-(5-Methyl-3-phenyl-1H-indol-2-yl)-1,2,4-oxadiazole* (**8b**). Obtained as a yellow solid (65%); mp ~124 °C; IR (KBr): *υ* = 3100 (aromatic CH) cm^–1^; ^1^H-NMR: *δ* = 2.69 (s, 3H, CH_3_), 6.72–7.28 (m, 9H, aromatic), 10.1 (s, 1H, NH, imidazole); ^13^C-NMR: *δ* = 36.3 (CH_3_), 110.6, 112.7, 119.3, 121.7. 122.6, 122.9, 123.9, 127.4, 128.9, 129.6, 131.2, 132.6, 134.8 142.6 (aromatic carbons); MS, *m/z* (%): 275.1 (M^+^, 83), 77 (100); Anal. Calcd. for C_17_H_13_N_3_O: C, 74.17; H, 4.76; N, 15.26. Found: C, 74.09; H, 4.66; N, 15.13.

### 3.4. Synthesis of 1,2,3-Triazole Derivatives ***10a**,**b***

A mixture of **4a**,**b** (10 mmol), hydroxylamine hydrochloride (0.69 g, 10 mmol), and sodium acetate (3 g, 25 mmol) was dissolved in ethanol (25 mL). The mixture was refluxed for 4 h. The reaction mixture was poured into ice/water with stirring while a yellow solid separated and was then collected by filtration. The crude product, so formed, was then refluxed in DMF for 5 h and the reaction mixture was poured into cold water. The products **10a**,**b** were purified by crystallization from ethanol.

*5-Benzyl-2-(4-chlorophenyl)-2H-1,2,3-triazol-4-amine* (**10a**). It was obtained as a yellow solid (75%); mp >250 °C; IR (KBr): *υ* = 3330 (br. NH_2_) cm^–1^; ^1^H-NMR: *δ* = 3.65 (s, 2H, CH_2_), 6.87 (s, 2H, NH_2_), 7.01–7.23 (m, 9H, aromatic); ^13^C-NMR: *δ* = 33.1 (CH_2_), 104.8, 121.3, 122.7, 122.9, 125.7, 128.3, 129.1, 132.0, 134.6, 141.0 (aromatic carbon); MS, *m/z* (%): 284.08 (M^+^, 65), 77 (84); Anal. Calcd. for C_15_H_13_ClN_4_: C, 63.27; H, 4.60; Cl, 12.45; N, 19.68. Found: C, 63.18; H, 4.53; Cl, 12.34; N, 19.62.

*5-Benzyl-2-(4-tolyl)-2H-1,2,3-triazol-4-amine* (**10b**). It was obtained as a yellow solid (70%); mp ~197 °C; IR (KBr): *υ* = 3340 (br. NH_2_) cm^–1^; ^1^H-NMR: *δ* = 2.44 (s, 3H, CH_3_), 3.61 (s, 2H, CH_2_), 5.82 (s, 2H, NH_2_), 7.01–7.24 (m, 9H, aromatic); ^13^C-NMR: *δ* = 30.8 (CH_3_), 32.6 (CH_2_), 106.4, 119.6, 121.2, 122.7, 124.3, 128.1, 128.4, 131.8, 133.2, 139.1 (aromatic carbons); MS, *m/z* (%): 264.14 (M^+^, 46), 77 (100); Anal. Calcd. for C_16_H_16_N_4_: C, 72.70; H, 6.10; N, 21.20. Found: C, 72.57; H, 6.02; N, 21.13.

### 3.5. Cyclization of ***4a**,**b*** with Chloroacetonitrile in the Presence of Et_3_N

A mixture of **4a**,**b** (10 mmol), chloroacetonitrile (0.75 g, 10 mmol), and triethylamine (0.5 mL) was irradiated at 80 W for 5 min (final temperature 140 °C). The reaction mixture was poured into a HCl/water mixture and the solid product, so formed, was then collected by filtration and recrystallized from ethanol.

*4-Amino-3-benzyl-1-(4-chlorophenyl)-1H-pyrazole-5-carbonitrile* (**12a**). This compound was obtained as a yellow solid (67%); mp ~227 °C; IR (KBr): *υ* = 3350 (br. NH_2_), 2210 (CN) cm^–1^; ^1^H-NMR: *δ* = 3.86 (s, 2H, CH_2_), 6.87 (s, 2H, NH_2_), 7.01–7.26 (m, 9H, aromatic); ^13^C-NMR: *δ* = 31.6 (CH_2_), 117.8 (CN), 119.9, 121.5, 122.3, 125.1, 125.4, 127.2, 129.8, 132.6, 133.8, 135.1, 138.7 (aromatic carbons); MS, *m/z* (%): 308.08 (M^+^, 27), 77 (100); Anal. Calcd. for C_17_H_13_ClN_4_: C, 66.13; H, 4.24; Cl, 11.48; N, 18.15. Found: C, 66.04; H, 4.07; Cl, 11.33; N, 18.05.

*4-Amino-3-benzyl-1-p-tolyl-1H-pyrazole-5-carbonitrile* (**12b**). This compound was obtained as a yellow solid (70%); mp ~204 °C; IR (KBr): *υ* = 3330 (br. NH_2_), 2190 (CN) cm^–1^; ^1^H-NMR: *δ* = 2.27 (s, 3H, CH_3_), 3.62 (s, 2H, CH_2_), 6.41 (s, 2H, NH_2_), 6.94–7.21 (m, 9H, aromatic); ^13^C-NMR: *δ* = 35.9 (CH_3_), 38.1 (CH_2_), 117.7 (CN), 119.2, 121.1, 121.7, 124.2, 127.9, 128.6, 129.7, 131.2, 132.4, 134.0, 136.4 (aromatic carbons); MS, *m/z* (%): 288.14 (M^+^, 62), 77 (100); Anal. Calcd. for C_18_H_16_N_4_: C, 74.98; H, 5.59; N, 19.43. Found: C, 74.88; H, 5.47; N, 19.27.

### 3.6. Synthesis of Pyrazolopyrimidine Derivatives

A mixture of **12a**,**b** (10 mmol) and dimethylformamide dimethylacetal (1.8 g, 15 mmol) in dry xylene (50 mL) was refluxed for 6 h. The reaction mixture was cooled and then the product, so formed, was refluxed with ammonium acetate (1.54 g, 20 mmol) and glacial acetic acid (25 mL) for 4 h. The reaction mixture was cooled and treated with petroleum ether whereby a yellowish solid precipitated and was collected by filtration. The pure product was obtained by crystallized from ethanol.

*3-Benzyl-1-(4-chlorophenyl)-1H-pyrazolo[4,3-d]pyrimidin-7-amine* (**14a**). This compound was obtained as a yellow solid (72%); mp >250 °C; IR (KBr): *υ* = 3350 (br. NH_2_) cm^–1^; ^1^H-NMR: *δ* = 3.36 (s, 2H, CH_2_), 5.87 (s, 2H, NH_2_), 7.08–7.17 (m, 9H, aromatic), 8.98 (s, 1H, CH pyrimidine); ^13^C-NMR: *δ* = 34.6 (CH_2_), 104.7, 110.4, 114.0, 119.1, 121.4, 123.9, 124.2, 127.3, 128.7, 130.2, 131.7, 134.3, 139.7 (aromatic carbons); MS, *m/z* (%): 335.09 (M^+^, 48), 77 (100); Anal. Calcd. for C_18_H_14_ClN_5_: C, 64.38; H, 4.20; Cl, 10.56; N, 20.86. Found: C, 64.26; H, 4.06; Cl, 10.39; N, 20.67.

*3-Benzyl-1-p-tolyl-1H-pyrazolo[4,3-d]pyrimidin-7-amine* (**14b**). This compound was obtained as a yellow solid (65%); mp >250 °C; IR (KBr): *υ* = 3330 (br. NH_2_) cm^–1^; ^1^H-NMR: *δ* = 2.84 (s, 3H, CH_3_), 3.17 (s, 2H, CH_2_), 5.61 (s, 2H, NH_2_), 6.86–7.19 (m, 9H, aromatic), 8.62 (s, 1H, CH pyrimidine); ^13^C-NMR: *δ* = 36.4 (CH_3_), 38.6 (CH_2_), 105.2, 112.4, 119.1, 119.7, 122.1, 124.9, 128.2, 128.6, 130.9, 132.3, 134.6, 135.0, 137.8 (aromatic carbons); MS, *m/z* (%): 315.1 (M^+^, 53), 77 (100); Anal. Calcd. for C_19_H_17_N_5_: C, 72.36; H, 5.43; N, 22.21. Found: C, 72.25; H, 5.31; N, 22.08.

## 4. Conclusions

2-Arylhydrazono-3-propanenitriles are readily obtainable versatile intermediates in the syntheses of a diversity of heterocycles, especially indoles, pyrazoles, 1,2,3-triazole, and pyrazolo[4,3-*d*]-pyrimidines, thus proving the general scope of our newly reported findings on the reactions of 2-aryl-hydrazononitriles.
